# Barriers to HIV and sexual and reproductive health care for female sex workers in Tete, Mozambique: results from a cross-sectional survey and focus group discussions

**DOI:** 10.1186/s12889-016-3305-5

**Published:** 2016-07-20

**Authors:** Yves Lafort, Faustino Lessitala, Balthazar Candrinho, Letitia Greener, Ross Greener, Mags Beksinska, Jenni A. Smit, Matthew Chersich, Wim Delva

**Affiliations:** International Centre for Reproductive Health, Ghent University, Gent, Belgium; International Centre for Reproductive Health-Mozambique, Maputo, Mozambique; Direcção Provincial de Saúde de Tete, Tete, Mozambique; MatCH Research Unit (Maternal, Adolescent and Child Health Research Unit), Department of Obstetrics and Gynaecology, Faculty of Health Sciences, University of the Witwatersrand, Durban, South Africa; Discipline of Pharmaceutical Sciences, College of Health Sciences, University of KwaZulu-Natal, Durban, South Africa; Wits Reproductive Health and HIV Institute, Faculty of Health Sciences, University of the Witwatersrand, Johannesburg, South Africa; The South African DST/NRF Centre of Excellence in Epidemiological Modelling and Analysis (SACEMA), University of Stellenbosch, Stellenbosch, South Africa; Center for Statistics, Hasselt University, Diepenbeek, Belgium

**Keywords:** Care seeking, Female sex workers, HIV, Mixed methods, Mozambique, Sexual and reproductive health

## Abstract

**Background:**

In the context of an operational research project in Tete, Mozambique, use of, and barriers to, HIV and sexual and reproductive health (HIV/SRH) commodities and services for female sex workers (FSWs) were assessed as part of a baseline situational analysis.

**Methods:**

In a cross-sectional survey 311 FSWs were recruited using respondent driven sampling and interviewed face-to-face, and three focus group discussions were held with respectively 6 full-time Mozambican, 7 occasional Mozambican and 9 full-time Zimbabwean FSWs, to investigate use of, and barriers to, HIV/SRH care.

**Results:**

The cross-sectional survey showed that 71 % of FSWs used non-barrier contraception, 78 % sought care for their last sexually transmitted infection episode, 51 % of HIV-negative FSWs was tested for HIV in the last 6 months, 83 % of HIV-positive FSWs were in HIV care, 55 % sought help at a health facility for their last unwanted pregnancy and 48 % after sexual assault, and none was ever screened for cervical cancer. Local public health facilities were by far the most common place where care was sought, followed by an NGO-operated clinic targeting FSWs, and places outside the Tete area. In the focus group discussions, FSWs expressed dissatisfaction with the public health services, as a result of being asked for bribes, being badly attended by some care providers, stigmatisation and breaches of confidentiality. The service most lacking was said to be termination of unwanted pregnancies.

**Conclusions:**

The use of most HIV and SRH services is insufficient in this FSW population. The public health sector is the main provider, but access is hampered by several barriers. The reach of a FSW-specific NGO clinic is limited. Access to, and use of, HIV and SRH services should be improved by reducing barriers at public health facilities, broadening the range of services and expanding the reach of the targeted NGO clinic.

## Background

Female sex workers (FSWs) are among the most vulnerable for sexual health risks, as a result of having multiple partners and sexual contacts [1]. The prevalence of HIV and other sexually transmitted infections (STI) among FSWs is several fold the prevalence in the general population [2, 3], HPV infection and resulting cervical cancer are frequent [4, 5], unwanted pregnancies are common [6–8], and FSWs are often victims of sexual and other types of violence [9, 10]. FSWs’ access to appropriate health care is hampered by high mobility, possible illegal immigration status, unfamiliarity with, stigmatisation and discrimination at, and inconvenient opening hours of, the local general health services [11, 12].

Sex work is common in the adjacent cities of Tete and Moatize in Mozambique. The growing mining industry and the main transport route connecting Malawi to Zimbabwe and the port of Beira attracts travellers, migrant labour and sex workers. An enumeration exercise carried out in the Tete-Moatize area in 2008 counted 4,415 FSWs recruiting clients in establishments on a Friday night at the beginning of the month [13]. HIV prevalence among FSWs was 50 % in 2006 [14]. The area (from now on referred to as ‘Tete’) has 8 public health centres, 1 public hospital, and 4 private clinics, and in Moatize there is a stand-alone drop-in clinic for most-at-risk populations that offers information, education and communication, condoms, STI care, contraceptive services, and HIV testing services (HTS) during the evening (4–10 PM), and is therefore called the Night Clinic. It is operated by a non-governmental organisation, in collaboration with the district health department [13].

Tete is one of the 4 cities participating in the DIFFER project (Diagonal Interventions to Fast-Forward Enhanced Reproductive health), a 5-years operational research project that aims at improving access to HIV/SRH services for FSWs by a better linkage between interventions targeted at FSWs and the general health services [15]. It applies a methodological framework for health systems research, starting with a situation analysis that informed the development of a context-specific package of interventions to strengthen HIV/SRH service delivery to FSWs. These packages are then implemented and assessed for their feasibility, acceptability, effectiveness, cost-effectiveness and sustainability. The situational analysis was conducted during the first 18 months of the project and applied a convergent parallel mixed methods design, combining multiple qualitative and quantitative research methods [16, 17]. The objectives were (1) to assess what HIV/SRH services are available at each site and to what extent they are adapted to the needs of FSWs, and (2) to what degree do FSWs make use of the available HIV/SRH services and what are the main barriers. The current article presents the main findings of the second objective of the situational analysis, the use of, and barriers to, HIV/SRH services by female sex workers, in Tete. The findings on the availability and FSW-friendliness of the services are presented elsewhere. The findings from the intervention implementation will be presented at a later stage once the evaluation of the intervention has been completed.

## Methods

The extent of the use of HIV/SRH commodities and services by FSWs was assessed between August 2013 and April 2014 through a cross-sectional survey, using respondent-driven sampling. RDS is similar to snowballing, but corrects for the bias towards FSWs with large social networks through statistical adjustments [18]. RDS begins with the selection of “seeds” who are known members of the FSW population. The seeds are instructed to enrol a limited number of other FSWs from their social circle for the survey, who in turn enrol other FSWs, and so on.

Initially, 6 seeds were recruited of different nationality (Zimbabwean/Mozambican), place of residence (Moatize/Tete) and number of clients (low/medium/high number of clients) and respondents were requested to each invite 3 new participants using referral coupons. Because of low uptake, the number of seeds was later increased to 13. Participants were interviewed face-to-face using an electronic questionnaire (QDS™ Version 2.0) addressing the use of HIV/SRH services and commodities. Coupons were managed using RDS Coupon Manager Version 3.0. Interview data were stored in QDS Data Warehouse and merged with the coupon information. Prevalence estimates and confidence intervals of variables were produced adjusting for unequal probabilities of inclusion due to varying social network sizes and the similarities in characteristics of persons within their social networks, using the RDS analysis package of STATA version 12 (StataCorp). We used the ‘Volz-Heckathorn’ estimator for the calculation of the weighed proportions and bootstrapping for the confidence intervals, correcting for non-normal distribution [19].

In addition, focus group discussions (FGD) were held with Zimbabwean and Mozambican FSWs for whom sex work was their main occupation (‘full-time’ FSWs), and Mozambican FSWs for whom it was not (‘occasional’ FSWs). All Zimbabwean FSWs are full-time FSWs in Tete. Nine FSWs of each type were invited and 9 Zimbabwean, 6 full-time Mozambican and 7 occasional Mozambican FSWs accepted to participate. Using a semi-structured guide in English or Portuguese a moderator, assisted by a note taker, explored access to and experiences with SRH services and recommendations for improving access. The discussions were audio-recorded, transcribed and analysed using NVivo 10 (QSR International) independently by two researchers. The transcripts were thematically inductively coded according to SRH service use, barriers to SRH services, satisfaction with current services, needs for additional services, and recommendations to improve the services. Desire for additional services was explored using a simple preference ranking exercise.

## Results

### Cross-sectional survey

#### Socio-demographic and sex work characteristics (Table 1)

**Table 1:** Characteristics of surveyed FSWs by nationality (*N* = 311)

	Mozambican FSWs (*N* = 78)		Foreign FSWs (*N* = 233)	
	Median	Q1–Q3	Median	Q1–Q3
Age in years	23.5	20–28	30	25.5–34
Years in current residence	4	2–19	2	1–3
Number of sex partners in the past week	5.5	4–10	14	10–20
Years doing sex work	3	2–6	4	2–7

A total of 311 FSWs were recruited. The majority were foreign nationals, 68 % were Zimbabwean, 25 % Mozambican and 7 % of another nationality. The median age of Mozambican FSWs was lower (23.5 years) than of foreign FSWs (30 years). The median number of years living at their current residence was 4 and 2 years (Mozambican and foreign FSWs, respectively). Foreign FSWs had a median number of 15 different partners in the past week, and Mozambican FSWs 5. The median number of years engaging in sex work was 3 and 4 years for Mozambican and foreign FSWs, respectively.

#### Use of HIV and sexual and reproductive health services (Table 2)

**Table 2:** Use of HIV and sexual and reproductive health services (*N* = 311)

	*n*	%	RDS adjusted %	95 % CI
Contraception use (*N* = 253)^a^				
Used contraception method	218	86.2	85.5	79.2–91.5
Used condoms only	38	15.0	14.1	8.9–20.1
Used non-barrier contraception method	179	70.8	70.3	62.0–78.1
Unwanted pregnancy				
Had unwanted pregnancy in the past 5 years	20	6.4	7.6	2.8–14.1
Sought help at a health facility (*N* = 20)^b^	11	55.0 %	-	-
STI care				
Had STI symptoms in the past year	172	56.0	49.5	42.0–57.3
Sought care at a health facility (*N* = 172)^c^	134	77.9	80.2	72.5–87.3
HTS (*N* = 214)^d^				
Tested for HIV in the last 6 months	108	50.5	56.0	46.5–65.0
HIV care				
Reported last HIV test to be positive (*N* = 278)^e^	128	46.0	46.4	38.6–54.4
Currently in HIV care (*N* = 128)^f^	106	82.8	84.1	75.0–25.0
Currently on ART (*N* = 128)^f^	85	66.4	69.2	58.2–80.2
Cervical cancer screening				
Ever screened for cervical cancer	0	0.0	-	-
Sexual violence				
Victim of sexual violence in past year	42	15.7	13.5	8.6–19.2
Sought medical care (*N* = 42)^g^	20	47.6	-	-
Attendance at public health facilities				
Had difficulty getting health care (*N* = 121)^h^	18	14.9	11.5	5.2–19.0
Was refused a health services in the past 12 months	0	0.0	-	-
Discloses as FSW	157	49.5	45.9	39.0–53.8
Feels treated like everybody else	297	96.1	94.8	91.0–98.3
Feels shame or guilt because of being FSW	61	19.9	19.2	13.6–25.4

^a^ *N* = Excludes FSWs with a pregnancy wish or not able to conceive

^b^ *N* = FSWs who had an unwanted pregnancy in the past 5 years

^c^ *N* = FSWs who had STI symptoms in the past year

^d^ *N* = Excludes FSWs who had tested positive for HIV more than 6 months ago

^e^ *N* = Excludes FSWs who never tested for HIV

^f^ *N* = HIV positive FSWs

^g^ *N* = FSWs who were victim of sexual violence in past year

^h^ *N* = FSWs who sought medical care in the past year

The majority of FSWs (86 %) who did not want to become pregnant were using a contraception method: 15 % were using condoms only and 71 % a modern non-barrier contraception method (hormonal, IUD or female sterilisation), of which 64 % combined with condoms (dual protection). When asked what action they had taken, eleven of the 20 FSWs (55 %) who reported an unwanted pregnancy in the past 5 years, replied to have gone to a health facility and the remaining to have done nothing. Approximately half (56 %) had either an abnormal vaginal discharge or genital ulcers in the past year and 78 % of these (134/172) had sought care at a health facility. Half (51 %) of the FSWs, excluding those who had tested positive for HIV more than 6 months ago, had tested for HIV in the past 6 months. Forty six percent reported to be HIV positive and 83 % of these were enrolled in HIV care and 66 % had already initiated antiretroviral therapy. None of the FSWs had, to their knowledge, ever been screened for cervical cancer. Of the 42 FSWs (16 %) who had been victim of sexual assault in the past year, about half (48 %) had sought medical care.

Fifteen percent of those who had sought medical care in the past year, reported to have had difficulties in obtaining it, but none reported to have been refused a health service. Half of the FSWs (50 %) said they disclose that they are a FSW, the majority (96 %) felt they were treated like everybody else and 20 % had feelings of shame or guilt because of being a FSW, when visiting a public health facility.

#### Place where care sought and satisfaction with received services (Table 3)

**Table 3:** Place where care sought and satisfaction with received services, by type of service

	General health care (*N* = 277)^b^		Contraceptive services (*N* = 213)^c^		STI care (*N* = 134)^d^		HTS (*N* = 241)^e^		HIV care (*N* = 105)^f^	
	RDS adjusted %	95 % CI	RDS adjusted %	95 % CI	RDS adjusted %	95 % CI	RDS adjusted %	95 % CI	RDS adjusted %	95 % CI
Place where last time care was sought										
Public health facilities	78.0	71.7–84.0	37.5	29.2–47.1	66.5	55.8–75.7	42.8	34.3–51.0	60.8	48.2–71.9
Night clinic	16.8	11.7–22.0	30.1	21.4–38.8	24.5	16.4–34.2	16.1	9.6–23.9	0.0	-
Private clinics	1.8	0.5–3.5	(0.8)^a^	(0.1–0.5)	0.0	-	(0.8)^a^	(0.2–3.3)	0.0	-
Informal health sector	6.6	3.4–10.6	15.1	9.8–20.9	4.4	2.2–8.7	0.0	-	0.0	-
Community outreach	0.0	-	4.9	2.2–8.3	0.0	-	21.9	15.8–28.9	0.0	-
Outside Tete	1.9	0.1–4.6	12.8	7.9–18.6	1.4	0.3–4.5	18.0	12.5–24.0	39.0	27.6–50.1
Reason for choice of place										
Where I always go	-	-	65.1	55.4–73.9	66.3	55.9–75.9	47.8	39.1–55.2	69.1	58.5–79.2
Nearby	-	-	42.5	32.9–51.9	43.9	32.8–55.3	42.7	34.9–50.9	32.6	20.9–44.1
I was referred there	-	-	17.3	11.5–23.4	39.9	29.8–51.0	16.4	10.3–23.9	19.3	11.7–28.5
Shorter waiting times	-	-	5.3	2.4–9.1	2.8	1.6–6.6	9.1	4.7–14.4	4.5	1.6–10.5
Privacy	-	-	3.7	1.0–7.6	4.1	1.4–8.7	6.3	3.1–9.9	10.6	4.3–18.3
Friendly Health personnel	-	-	1.1	6.2	0.0	-	10.0	5.4–15.2	0.0	-
Cost is low or free	-	-	3.7	1.2–7.2	(2.5)^a^	(0.0–5.3)	4.4	1.7–7.7	2.2	0.4–5.0
Quality of care	-	-	0.7	0.2–2.0	0.0	-	2.5	0.7–4.9	4.5	1.3–10.3
Satisfaction with the received services										
Very satisfied	30.0	20.1–36.3	29.0	20.4–38.6	26.6	18.0–36.2	32.4	24.4–41.8	33.5	22.0–45.4
Satisfied	71.7	63.3–79.3	70.2	61.5–48.8	71.2	61.8–80.4	66.9	58.3–76.1	62.6	51.1–74.7
Little or not satisfied	0.3	0.3–1.2	0.9	0.5–2.8	2.2	0.7–5.9	(0.6)^a^	(0.1–4.0)	3.9	0.5–10.2

^a^Bootstrap analysis was not possible because of too few observations in some categories. A weighed proportion was calculated instead

^b^ *N* = Excludes FSWs who didn’t respond to the questions

^c^ *N* = FSWs who used a contraception method and responded to the questions

^d^ *N* = FSWs who sought care for last STI episode

^e^ *N* = FSWs who had an HIV test in the past 2 years

^f^ *N* = FSWs who were in HIV care and responded to the questions

The public health facilities of Tete were the most common source of care for all services, ranging from 38 % for contraceptive services to 78 % for general health care. The Night Clinic was an important provider of contraceptive services (32 %), STI care (25 %) and to a lesser extent general health care (17 %) and HTS (16 %). Community outreach was an important source of HTS (22 %). A third (39 %) of FSWs sought care for HIV and a fifth (18 %) were last tested for HIV outside Tete.

The most common reasons given for the choice of the place where they last went were that it is where they always go (ranging from 48 % for HTS to 69 % for HIV care), that it is nearby (ranging from 33 % to 44 %) and that they were referred there (ranging from 17 % to 40 %). When asked if they were satisfied with the services they had received the last time they sought care, very few FSWs reported to be not or little satisfied, ranging from 0.3 % for general health services to 3.9 % for HIV care.

Care seeking, the place where care is sought, the reasons for the choice and satisfaction with services did not substantially differ between Mozambican and foreign FSWs, or between FSWs who sought care at public health services and the Night Clinic.

### Focus group discussions

The median age of the 9 full-time Zimbabwean FSWs, 6 Mozambican full-time FSWs and 7 Mozambican occasional FSWs participating in the focus group discussions was 36, 23 and 22 years, respectively. The median number of sex partners in the past week was 15, 9.5 and 3, respectively, and the median number of years engaging in sex work 12, 4 and 3.

#### Need of additional HIV/SRH services

When asked what services were most in need, Zimbabwean FSWs listed most often termination of pregnancy (TOP). This service was also often listed by occasional Mozambican FSWs and 1 full-time Mozambican FSW. Care for incomplete miscarriages/abortions and services for victims of violence were also often mentioned. Other services listed included more information on certain SRH topics, better STI and HIV care, cervical cancer services, emergency contraception and female condoms.

#### HIV/SRH care seeking behaviour, barriers and satisfaction

Mozambican FSWs reported to primarily use the public health facilities for HIV/SRH care. The Night Clinic was known and used by most full time Mozambican FSWs, but most of the occasional FSWs did not know of its existence and one thought it was only intended for Zimbabwean FSWs. Among Zimbabwean FSWs it was known and used by those operating in Moatize. Zimbabwean FSWs reported to often use the services of ‘bush doctors’. These are illegal providers of modern medicine (oral and injectable medicines), either at the market or at the client’s home. Another source of care is the direct purchase of drugs at pharmacies.

The reasons for choosing a public health facility was mostly because it was nearby, but satisfaction with the services was generally very low. The most common reason for dissatisfaction, by all groups of FSWs, was the common practice of asking for a bribe by health providers. If you were willing to pay a bribe, you were rapidly and well attended. But if not, you were either put at the end of the queue, with the risk of not being attended that day, or completely refused the service. This was also the case for services that are supposed to be free and life-saving, such as antiretroviral therapy (ART).

*‘(translated from Portuguese) … for me it is the same. Without money I can’t get any services. You stay in line and they can send you back home. But with money, all is easy. You can be the first to be attended.’ (Mozambican occasional SW)*

Another important complaint was the bad reception by staff, especially if they were recognised as a sex worker and/or refused to pay bribes. This included sometimes refusal to offer services. In particular female providers were said to treat sex workers badly, saying that they were stealing their husbands. The younger occasional Mozambican FSWs reported that they were refused contraceptive services because they were too young. STI care was sometimes refused because of not bringing their partner. It was agreed though that reception by some other providers is very good.

*‘(translated from Portuguese) …Me to… I even was beaten, up to been called ‘whore, get out of here, or you might seduce our husbands’… (Mozambican full-time FSW)*

*‘I usually go to = X = when I have problems. There is one person I do not see anything bad in his service. I always get a good service.’ (Zimbabwean FSW)*

A third common reason for dissatisfaction, and reason for not using the public services at all, is the lack of privacy and confidentiality. This included fear of being recognised by other users, including their clients and other FSWs, while attending certain services, in particular HIV care; lack of privacy during the consultation; and breaches of confidentiality by the providers. Some FSWs also expressed a concern to be recognised when attending the Night Clinic. Some FSWs do not go for ART out of fear that their partner might discover they are HIV positive.

*‘For example at = X = hospital, I have sworn never to set my foot there again. Because if I explain my problem to someone, that my vagina is itchy they start telling someone else or another nurse in Portuguese, then start laughing someone else comes to peep at me, I will never see that they are good at their work. In that case ah it is better to go to the Bush Doctors where it will be just the two of us at home.’ (Zimbabwean SW)*

Other reasons for dissatisfaction were the common drugs shortages, the long waiting times and lengthy lunch breaks, the lack of information and explanations given by providers, and the short consultation time.

Dissatisfaction with the public services was greatest among Zimbabwean FSWs. Because of being foreigners and therefore more easily identifiable as FSWs they are requested to pay higher bribes than Mozambicans and suffer more from bad reception and discrimination. An additional barrier is the language, with few of the health staff speaking Shona or English. Level of dissatisfaction was lowest among occasional FSWs, because they were not recognised as FSW and therefore less discriminated.

*‘…there is this woman hii.., she does not even listen, she does not care because the moment she sees a ‘Zimbabweana’ [Zimbabwean] she just says puta [prostitute] Oh my God our husbands are gone!…’ (Zimbabwean SW)*

The FSWs that had used the Night Clinic were highly satisfied with the services mostly because they were well received, the chances to be seen by someone they knew were less, there are fewer clients and the service is fast, and the necessary drugs are always available. Other reasons were that more information is provided, the opening times are convenient, there is no discrimination, the care is better (includes clinical examination and injection) and is free of charge.

*‘I agree with = X=, If you go to the private clinic (=Night Clinic=) they have time for you to ask you to lay down and do an examination. If they give you an injection you feel that....yes… you have been treated really.’ (Zimbabwean SW)*

Zimbabwean FSWs discussed the topic of legalisation of TOP, with most being in favour, but some heavily opposed stating that it would be used as a contraception method. All agreed that post-abortion care needs to be improved and that ‘uterus cleaning’ should be done. With uterus cleaning they meant evacuation through curettage or aspiration. This service was said to be currently not offered.

#### Recommendations to improve health services

When asked what could be done to improve access to services and how they felt about a separate clinic for most-at-risk women, most sex workers were strongly in favour of maintaining the Night Clinic and expanding it to Tete City. Some however were not in favour because of the risk of stigmatisation when you are seen entering the clinic. The consensus was that it was necessary to maintain and expand the Night Clinic, but also improve access to the public services.

## Discussion

We assessed the use of different HIV and sexual and reproductive health commodities and services combining two complementary methods. The cross-sectional survey revealed that the use of HIV and SRH services is often insufficient, with high proportions of FSWs not using a service or commodity they need, but it was not able to identify what the barriers to use are. Few respondents reported difficulties in getting health care and when asked how they feel treated almost all said like everyone else. Very few reported to be dissatisfied with the received services. The only indication of fear of stigmatisation was that half of the FSWs admitted they did not disclose to be a FSW. This is in contrast with the results of the focus group discussions where participants expressed great dissatisfaction with the services received at public health facilities.

Being asked for bribes by providers was the most common reason for dissatisfaction, although this is not specific to FSWs but a general practice in the Mozambican public health sector [20]. It is related to factors, such as the poor economic situation of the country, that are beyond the scope of a FSW intervention as ours. Also breaches of confidentiality by the providers is not unique to FSWs. Bad attendance, at least by some providers, was however strongly perceived to be related to being a FSW, as was the fear of being recognised as a FSW by other users. This is consistent with what has been described elsewhere [11, 21, 22]. A better reception and confidentiality can be addressed by sensitising and training providers in FSW-friendly services, but fear of stigmatisation by other users is harder to solve.

Those who had used the Night Clinic clearly preferred it above the public sector. The attendance and the perceived quality of the services is much better, and the fear of stigmatisation is less, although still possible if seen entering the clinic. This is again similar to what was found elsewhere in Africa [11, 23].

Despite being disliked, the public health sector was by far the main provider of HIV/SRH services and the reach of the Night Clinic was limited. The reasons given for choosing the place of care were mostly practical, such as that it is nearby, that it is ‘where I always go’ or that someone had referred them there. Aspects of quality of care were rarely given as motive. The main reasons why the Night Clinic is still not commonly used are probably that it is located too far from where most FSWs reside and that it is still not widely known, in particular by Mozambican FSWs and FSWs of Tete city.

An important finding was that many FSWs use some HIV/SRH services outside Tete, mostly in their place of origin. The reasons for this are not known. Possible explanations are that FSWs frequently travel to their home area and that they are more acquainted with the services or less stigmatised and discriminated there. This has implications for interventions that aim to improve access to care, in particular HIV care, and it needs to be assessed how linkage with outside services can be improved. The link between mobility and poor retention in HIV care has been well documented but effective strategies to tackle the problem are still lacking [2, 24].

The HIV/SRH services offered at the public health facilities and the Night Clinic included contraceptive services, STI care, HTS and HIV care. Services for victims of sexual and gender-based violence did not yet exist; cervical cancer screening was in the process of being introduced and still not widely available; and termination of pregnancy (TOP) was still illegal and only available in exceptional cases at the provincial hospital. Of these services, TOP was the one most mentioned by FSWs in the FGDs as lacking, together with care for post-abortion complications. Mozambique has recently legalised TOP and access for FSWs should be ensured as soon as it becomes available.

We combined two complementary methods, each with their strengths and limitations. A cross-sectional survey has the advantage to reach a larger and representative sample of the target population, but does not allow for deeper exploration of the responses given. It is also prone to recollection bias, poor understanding of the question, social desirability bias, or reluctance to divulge sensitive personal information [25]. This probably explains why few FSWs expressed dissatisfaction with the public health services in the cross-sectional survey. An RDS approach facilitates reaching less visible FSWs, but it assumes successful recruitment of participants by their peers. In our study, refusal rate among occasional Mozambican FSWs was high and we believe that they might be under-represented. Focus group discussions allow a more in-depth exploration of the responses and facilitate a more natural discussion, but the representativeness of the participants is not assured and responses can be driven by the more outspoken participants [26]. It is therefore unsurprising that the results of FGDs appear at first sight contradictory with the cross-sectional survey, despite the socio-demographic characteristics of the participants being similar. We argue that rather than viewing these differences a limitation; they confirm the importance of using a mixed-methods approach, yielding complementary results leading to an integrated conclusion [16, 27].

## Conclusion

The use of HIV and SRH services is still insufficient in this female sex worker population. The public health sector is the main provider, but access is hampered by barriers, such as asking for bribes, bad attendance, stigmatisation and breaches of confidentiality. The services at an FSW-specific NGO clinic are more appreciated, but its reach is limited. In a next step, access to, and use of, HIV and SRH services should be improved by reducing the barriers at public health facilities, broadening the range of services offered and expanding the reach by the targeted clinic.

## Abbreviations

ART, antiretroviral therapy; DIFFER, diagonal interventions to fast-forward expanded reproductive health; FSW, female sex worker; HIV, human immunodeficiency virus; HTS, HIV testing services; IUD, intra-uterine device; NGO, Non-Governmental Organisation; RDS, respondent driven Sampling; SRH, sexual and reproductive health; STI, sexually transmitted infections; TOP, termination of pregnancy
